# Supplementary material for: MME+ fibro-adipogenic progenitors are the dominant adipogenic population during fatty infiltration in human skeletal muscle
Source: Commun Biol. 2023 Jan 27;6:111. doi: 10.1038/s42003-023-04504-y (PMC9883500; doi:10.1038/s42003-023-04504-y)
Supplement: Supplementary file 2 — Supplementary Information [file 42003_2023_4504_MOESM2_ESM.pdf]

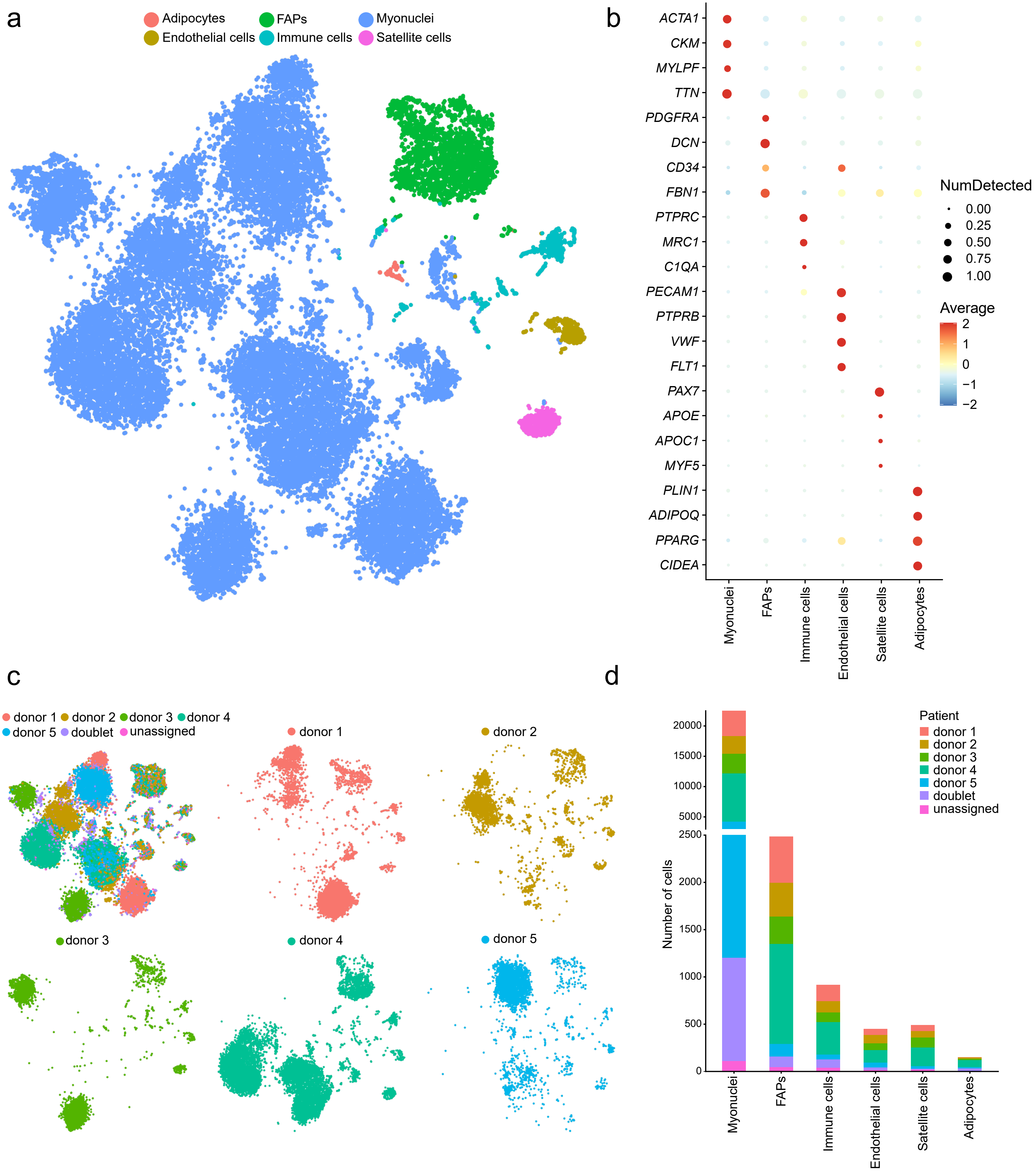

a
Adipocytes
FAPs
Myonuclei
Endothelial cells
Immune cells
Satellite cells
b
ACTA1
CKM
MYLPF
TTN
PDGFRA
DCN
CD34
FBN1
PTPRC
MRC1
C1QA
PECAM1
PTPRB
VWF
FLT1
PAX7
APOE
APOC1
MYF5
PLIN1
ADIPOQ
PPARG
CIDEA
NumDetected
0.00
0.25
0.50
0.75
1.00
Average
2
1
0
−1
−2
Myonuclei
FAPs
Immune cells
Endothelial cells
Satellite cells
Adipocytes
c
donor 1
donor 2
donor 3
donor 4
donor 5
doublet
unassigned
d
20000
15000
10000
5000
2500
2000
1500
1000
500
0
Number of cells
Patient
donor 1
donor 2
donor 3
donor 4
donor 5
doublet
unassigned
Myonuclei
FAPs
Immune cells
Endothelial cells
Satellite cells
Adipocytes

Supplementary Fig. 1: snRNA-seq analysis from human muscle identifies main cell populations. Related to Fig. 1.

a TSNE plot of main cell types from human muscle, color-coded by the identified populations. **b** Dot plot of centered logcounts values from cell type-specific marker genes. Color and size of the dots indicate the centered logcount value and the proportion of cells that express the gene, respectively. **c** TSNE plots of full (upper-left) and per-patient (upper-middle, upper-right and bottom panels) snRNA-seq datasets, color-coded by the patient of origin. **d** Stacked bar plots showing the contribution of each human patient to each cluster. Color-coded by the patient of origin.

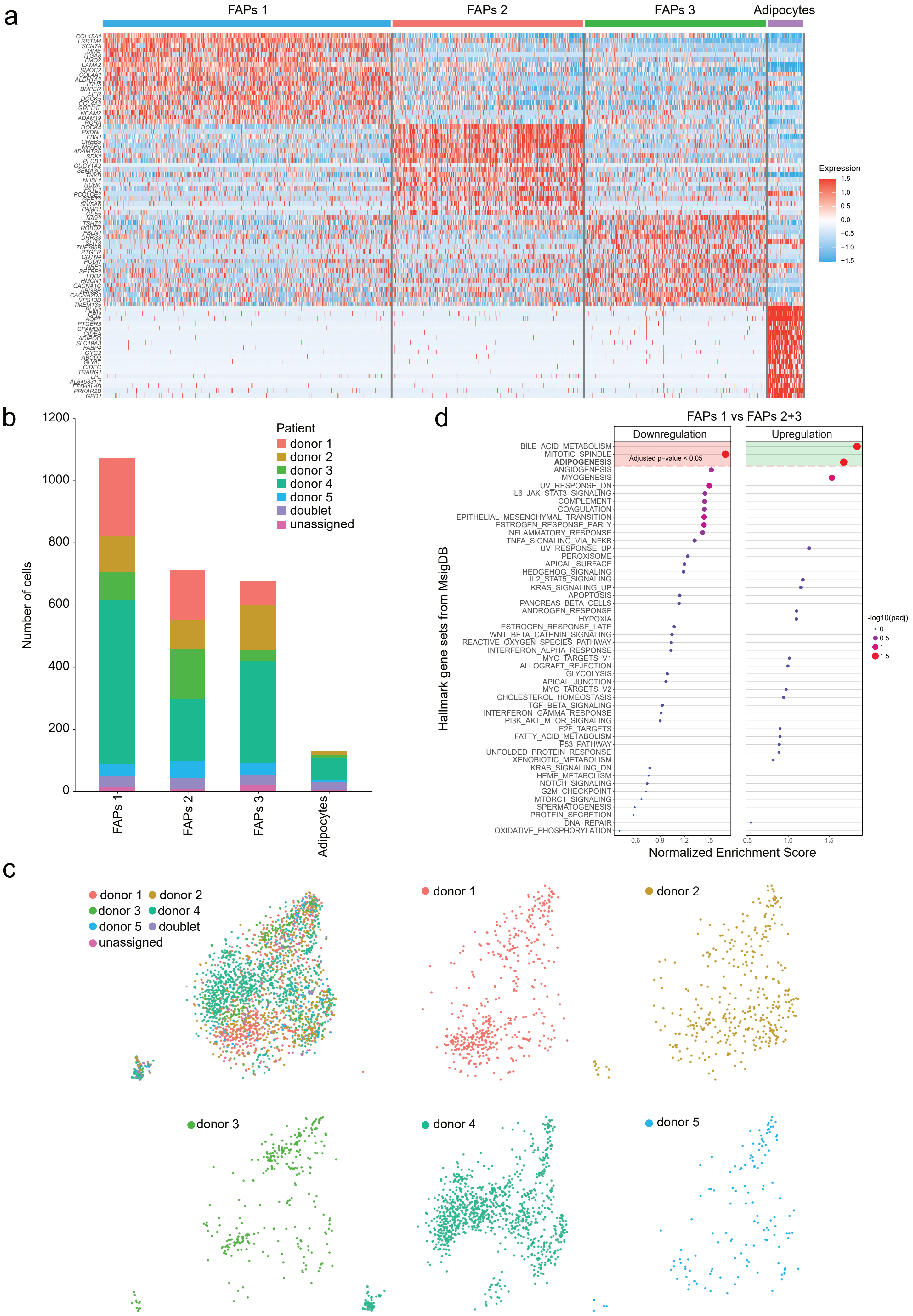
a
FAPs 1
FAPs 2
FAPs 3
Adipocytes
COL15A1
LRRTM4
SCN7A
MME
ITGA8
FMO2
LAMA2
SMOC2
COL4A1
ALDH1A2
ITIH5
BMPER
LIFR
DOCK5
COL4A2
GREB1L
NCAM2
ADAM19
RORA
DOCK4
PXDNL
FBN1
CREB5
MFAP5
ADAMTS5
SDK1
PLCB1
GUCY1A2
SEMA3C
TNXB
NHSL1
HUNK
FSTL1
PCOLCE2
GFPT2
SHISA6
PAMR1
CD55
NAV2
TSHZ2
ROBO2
FBLN1
DHRS3
SLIT3
ZNF385B
PTGFR
CNTN4
PODN
NRP1
SETBP1
LDB2
HMCN1
CACNA1C
ABI3BP
CACNA2D3
VPS13D
TMEM135
PLIN1
CPM
AQP7
PTGER3
CPAMD8
CIDEA
ADIPOQ
SLC19A3
FABP4
GYG2
ABCD2
GLYAT
CIDEC
TRARG1
LPL
AL845331.1
EPB41L4B
PRKAR2B
GPD1
Expression
1.5
1.0
0.5
0.0
−0.5
−1.0
−1.5
b
Number of cells
1200
1000
800
600
400
200
0
Patient
donor 1
donor 2
donor 3
donor 4
donor 5
doublet
unassigned
FAPs 1
FAPs 2
FAPs 3
Adipocytes
d
FAPs 1 vs FAPs 2+3
Downregulation
Upregulation
Adjusted p−value < 0.05
Hallmark gene sets from MsigDB
BILE_ACID_METABOLISM
MITOTIC_SPINDLE
ADIPOGENESIS
ANGIOGENESIS
MYOGENESIS
UV_RESPONSE_DN
IL6_JAK_STAT3_SIGNALING
COMPLEMENT
COAGULATION
EPITHELIAL_MESENCHYMAL_TRANSITION
ESTROGEN_RESPONSE_EARLY
INFLAMMATORY_RESPONSE
TNFA_SIGNALING_VIA_NFKB
UV_RESPONSE_UP
PEROXISOME
APICAL_SURFACE
HEDGEHOG_SIGNALING
IL2_STAT5_SIGNALING
KRAS_SIGNALING_UP
APOPTOSIS
PANCREAS_BETA_CELLS
ANDROGEN_RESPONSE
HYPOXIA
ESTROGEN_RESPONSE_LATE
WNT_BETA_CATENIN_SIGNALING
REACTIVE_OXYGEN_SPECIES_PATHWAY
INTERFERON_ALPHA_RESPONSE
MYC_TARGETS_V1
ALLOGRAFT_REJECTION
GLYCOLYSIS
APICAL_JUNCTION
MYC_TARGETS_V2
CHOLESTEROL_HOMEOSTASIS
TGF_BETA_SIGNALING
INTERFERON_GAMMA_RESPONSE
PI3K_AKT_MTOR_SIGNALING
E2F_TARGETS
FATTY_ACID_METABOLISM
P53_PATHWAY
UNFOLDED_PROTEIN_RESPONSE
XENOBIOTIC_METABOLISM
KRAS_SIGNALING_DN
HEME_METABOLISM
NOTCH_SIGNALING
G2M_CHECKPOINT
MTORC1_SIGNALING
SPERMATOGENESIS
PROTEIN_SECRETION
DNA_REPAIR
OXIDATIVE_PHOSPHORYLATION
0.6
0.9
1.2
1.5
0.5
1.0
1.5
Normalized Enrichment Score
-log10(padj)
0
0.5
1
1.5
c
donor 1
donor 2
donor 3
donor 4
donor 5
doublet
unassigned
donor 1
donor 2
donor 3
donor 4
donor 5

Supplementary Fig. 2: snRNA-seq analysis from human muscle identifies 3 FAPs populations. Related to Fig. 1.

a Heatmap with Z-scores showing top enriched marker genes per cell type. Each row represents a gene and each column a cell. Columns are ordered and color-coded based on the cluster of origin. **b** Stacked bar plots showing the contribution of each human patient to each cluster. Color-coded by thepatient of origin. **c** TSNE plots of full (upper-left) and per-patient (upper-middle, upper-right and bottom panels) snRNA-seq datasets, color-coded by the patient of origin. **d** Dot plots showing downregulated (left panel) or upregulated (right panel) pathways from GSEA of FAPs 1 compared to FAPs 2+3. X-axis indicates the normalized enrichment score (NES) for each pathway. Color and size of the dots indicate adjusted p-values (FDR). Significant values are delimited by the red dashed line (Adjusted p-value < 0.05 or -log10(adjusted p-value) > 1.3).

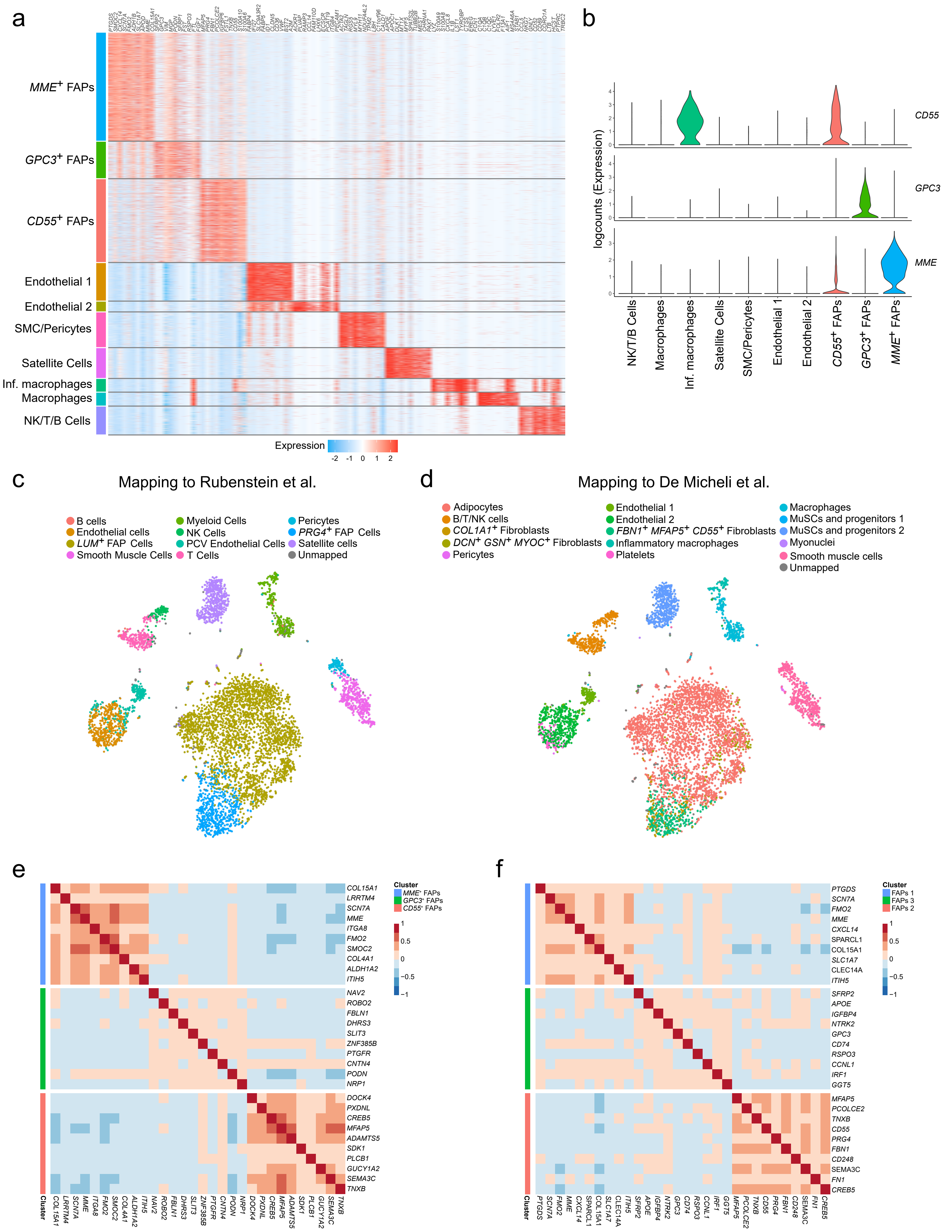
a
MME+ FAPs
GPC3+ FAPs
CD55+ FAPs
Endothelial 1
Endothelial 2
SMC/Pericytes
Satellite Cells
Inf. macrophages
Macrophages
NK/T/B Cells
Expression
-2 -1 0 1 2
b
logcounts (Expression)
CD55
GPC3
MME
NK/T/B Cells
Macrophages
Inf. macrophages
Satellite Cells
SMC/Pericytes
Endothelial 1
Endothelial 2
CD55+ FAPs
GPC3+ FAPs
MME+ FAPs
c
Mapping to Rubenstein et al.
B cells
Endothelial cells
LUM+ FAP Cells
Smooth Muscle Cells
Myeloid Cells
NK Cells
PCV Endothelial Cells
T Cells
Pericytes
PRG4+ FAP Cells
Satellite cells
Unmapped
d
Mapping to De Micheli et al.
Adipocytes
B/T/NK cells
COL1A1+ Fibroblasts
DCN+ GSN+ MYOC+ Fibroblasts
Pericytes
Endothelial 1
Endothelial 2
FBN1+ MFAP5+ CD55+ Fibroblasts
Inflammatory macrophages
Platelets
Macrophages
MuSCs and progenitors 1
MuSCs and progenitors 2
Myonuclei
Smooth muscle cells
Unmapped
e
Cluster
MME+ FAPs
GPC3+ FAPs
CD55+ FAPs
1
0.5
0
−0.5
−1
COL15A1
LRRTM4
SCN7A
MME
ITGA8
FMO2
SMOC2
COL4A1
ALDH1A2
ITIH5
NAV2
ROBO2
FBLN1
DHRS3
SLIT3
ZNF385B
PTGFR
CNTN4
PODN
NRP1
DOCK4
PXDNL
CREB5
MFAP5
ADAMTS5
SDK1
PLCB1
GUCY1A2
SEMA3C
TNXB
f
Cluster
FAPs 1
FAPs 3
FAPs 2
PTGDS
SCN7A
FMO2
MME
CXCL14
SPARCL1
COL15A1
SLC1A7
CLEC14A
ITIH5
SFRP2
APOE
IGFBP4
NTRK2
GPC3
CD74
RSPO3
CCNL1
IRF1
GGT5
MFAP5
PCOLCE2
TNXB
CD55
PRG4
FBN1
CD248
SEMA3C
FN1
CREB5

Supplementary Fig. 3: scRNA-seq analysis from human muscle identifies main FAPs and mononuclear cell populations. Related to Fig. 2.

a Heatmap with Z-scores showing top enriched marker genes per cell type. Each column represents a gene and each row a cell. Rows are ordered and color-coded based on the cluster of origin. **b** Violin plots of logcounts values from marker genes naming each FAP subpopulation, color-coded by the identified subpopulations. **c** TSNE plot of main cell populations identified in human muscle by the automatic mapping to Rubenstein et al. dataset, color-coded by the identified populations. **d** Same as **c** but mapping to De Micheli et al. dataset. **e** Heatmap representing Pearson's correlation values of human FAP markers from the snRNA-seq dataset in the human FAPs scRNA-seq dataset. Horizontal color-coded separation indicates the set of marker genes from each FAP subpopulation. **f** Heatmap representing Pearson's correlation values of human FAP markers from the scRNA-seq dataset in the human FAPs snRNA-seq dataset. Horizontal color-coded separation indicates the set of marker genes from each FAP subpopulation.

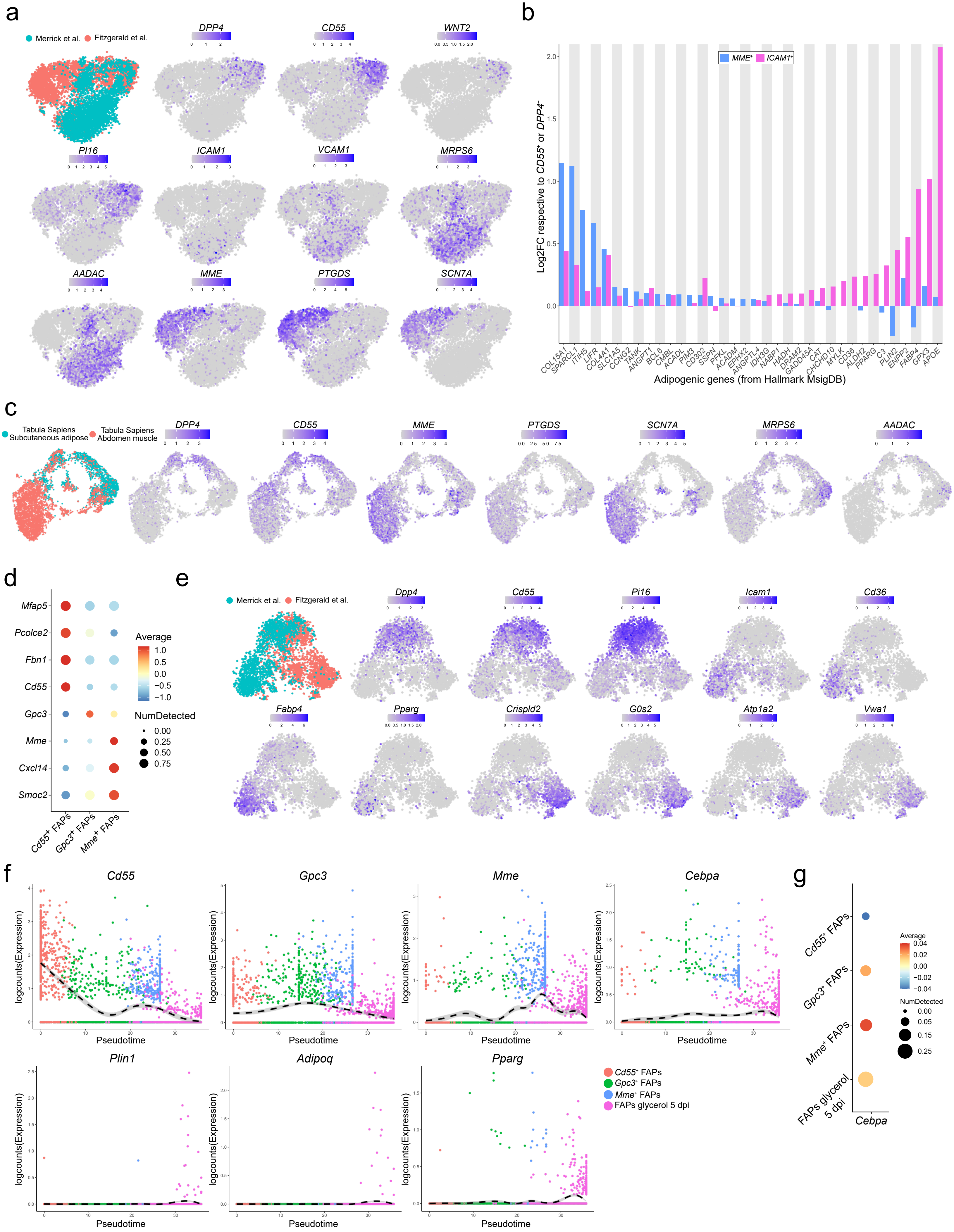
a
Merrick et al.
Fitzgerald et al.
DPP4
CD55
WNT2
PI16
ICAM1
VCAM1
MRPS6
AADAC
MME
PTGDS
SCN7A
b
MME+
ICAM1+
Log2FC respective to CD55+ or DPP4+
Adipogenic genes (from Hallmark MsigDB)
COL15A1
SPARCL1
ITIH5
LIFR
COL4A1
SLC1A5
CCNG2
TANK
ANGPT1
BCL6
CMBL
ACADL
PIM3
CD302
SSPN
PFKL
ACADM
EPHX2
ANGPTL4
IDH3G
NABP1
HADH
DRAM2
GADD45A
CAT
CHCHD10
MYLK
CD36
ALDH2
PPARG
C3
PLIN2
ENPP2
FABP4
GPX3
APOE
c
Tabula Sapiens Subcutaneous adipose
Tabula Sapiens Abdomen muscle
DPP4
CD55
MME
PTGDS
SCN7A
MRPS6
AADAC
d
Mfap5
Pcolce2
Fbn1
Cd55
Gpc3
Mme
Cxcl14
Smoc2
Average
NumDetected
Cd55+ FAPs
Gpc3+ FAPs
Mme+ FAPs
e
Merrick et al.
Fitzgerald et al.
Dpp4
Cd55
Pi16
Icam1
Cd36
Fabp4
Pparg
Crispld2
G0s2
Atp1a2
Vwa1
f
Cd55
Gpc3
Mme
Cebpa
Plin1
Adipoq
Pparg
logcounts(Expression)
Pseudotime
Cd55+ FAPs
Gpc3+ FAPs
Mme+ FAPs
FAPs glycerol 5 dpi
g
Cd55+ FAPs
Gpc3+ FAPs
Mme+ FAPs
FAPs glycerol 5 dpi
Cebpa
Average
NumDetected

Supplementary Fig. 4: *MME*⁺ and *GPC3*⁺ muscle FAPs are not found in adipose tissue. Related to Fig. 3.

a TSNEplots of FAPs marker genes identified in the integration of human muscle FAPs and human adipose progenitor cells from Merrick et al., color-coded by logcounts values. **b** Log2-FoldChange of adipogenic genes (from Hallmark MsigDB) found to be upregulated in *MME*$^+$ or *ICAM1*$^+$ cells. Each log2-FoldChange is relative to *CD55*$^+$ or *DPP4*$^+$ for *MME*$^+$ and *ICAM1*$^+$ cells respectively. **c** Same as **a** but UMAP plots showing FAPs marker genes identified in the integration of human abdomen muscle and human subcutaneous adipose tissue datasets from Tabula Sapiens, color-coded by logcounts values. **d** Dot plot of centered logcounts values from identified marker genes in each mouse FAP subpopulations. Color and size of the dots indicate the centered logcount value and the proportion of cells that express the gene, respectively. **e** Same as **a** but showing FAPs marker genes identified in the integration of mouse muscle FAPs and adult mouse (10 weeks) adipose progenitor cells from Merrick et al., color-coded by logcounts values. **f** Scatter plots showing logcounts values (Y-axis) of genes along the pseudotime trajectory (X-axis) from the integration of basal mouse muscle FAPs withFAPs 5 days after glycerol injection from Xu *et al.*, color-coded by the different FAPs populations. Dashed line indicates the smoothed conditional mean of logcount values along the pseudotime axis. **g** Dot plot of centered logcounts values from *Cebpa* in each mouse FAP subpopulations from the integration between basal mouse muscle FAPs and FAPs 5 days after glycerol injection from Xu *et al.* Color and size of the dots indicate the centered logcount value and the proportion of cells that express the gene.

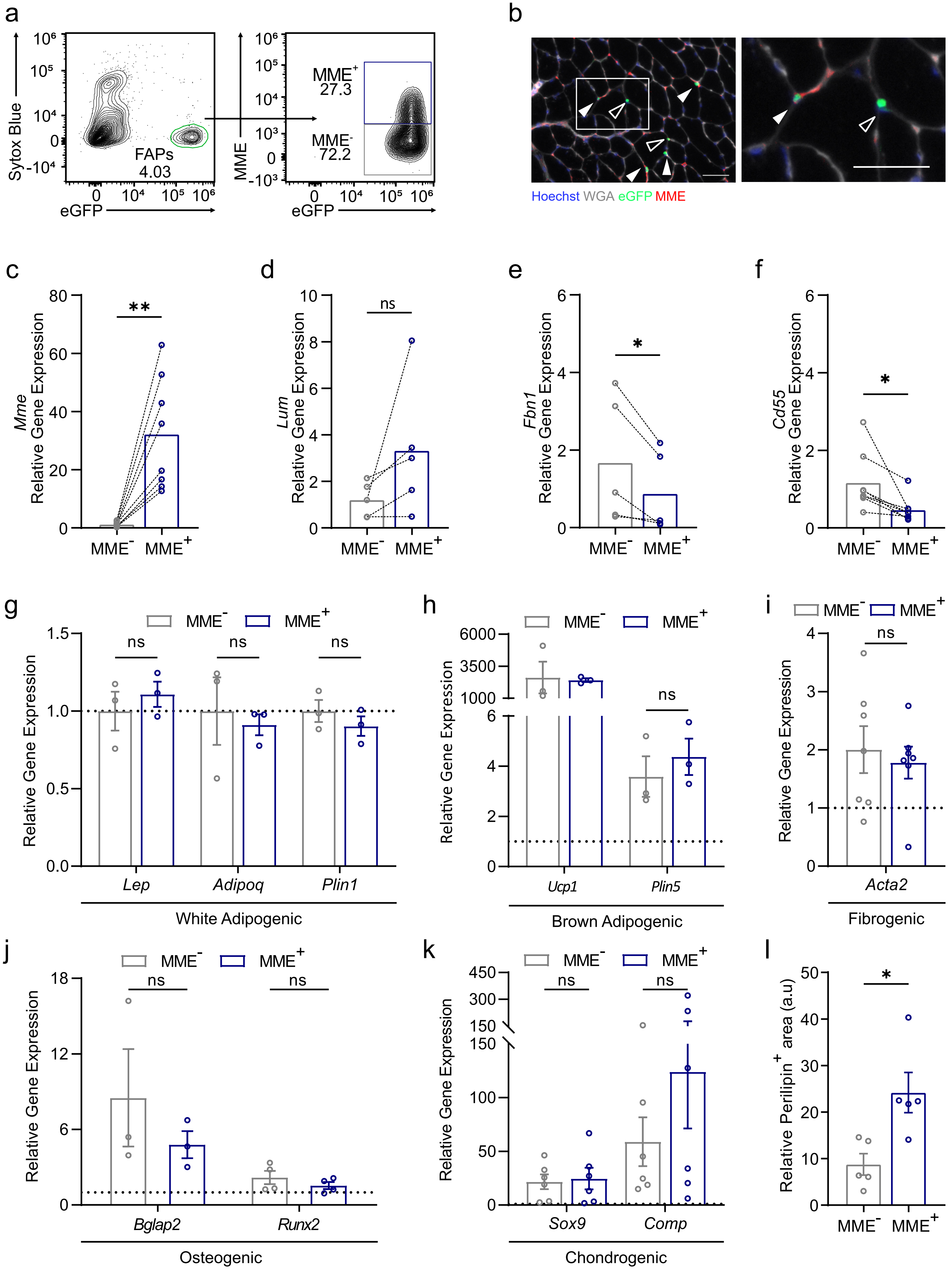

a
Sytox Blue
FAPs
4.03
eGFP
MME
MME+
27.3
MME-
72.2
b
Hoechst WGA eGFP MME
c
Mme
Relative Gene Expression
**
MME- MME+
d
Lum
Relative Gene Expression
ns
e
Fbn1
Relative Gene Expression
*
f
Cd55
Relative Gene Expression
*
g
MME- MME+
Relative Gene Expression
ns
Lep
Adipoq
Plin1
White Adipogenic
h
Ucp1
Plin5
Brown Adipogenic
i
Acta2
Fibrogenic
j
Bglap2
Runx2
Osteogenic
k
Sox9
Comp
Chondrogenic
l
Relative Perilipin+ area (a.u)

Supplementary Fig 5: MME$^+$ are a highly adipogenic fraction of PDGFRα^+ FAPs. Related to Fig. 4.

a Representative FACS plot outlining the sorting strategy to isolate PDGFRα-eGFP$^+$MME$^{-/+}$ FAPs. **b** Immunofluorescent staining of MME on muscle sections from PDGFRα-eGFP mice demonstrating the presence of both MME$^+$ (full white arrows) and MME$^-$ (white outlined arrows) FAPs. (green, eGFP$^+$; grey, WGA$^+$, red, MME$^+$, blue, Hoechst$^+$ Scale Bar: 50um). **c** Relative expression of *Mme* in freshly sorted MME$^{-/+}$ FAPs. **d** Relative expression of *Lum* in freshly sorted MME$^{-/+}$ FAPs. **e** Relative expression of *Fbn1* in freshly sorted MME$^{-/+}$ FAPs. **f** Relative expression of *Cd55* in freshly sorted MME$^{-/+}$ FAPs. **g** Relative gene expression of *Lep, Adipoq,* and *Plin1* in MME$^{-/+}$ FAP derived adipocytesdifferentiated in full white adipogenic medium. Related to Fig. 4e. **h** Relative gene expression of *Ucp1* and *Plin5* in MME$^{-/+}$ FAPs derived brown adipocytes. **i** Relative gene expression of *Acta2* in MME$^{-/+}$ FAP derived fibroblasts differentiated in fibrogenic medium. **j** Relative gene expression of *Bglap2* and *Runx2* in MME$^{-/+}$ FAP derived osteocytes. **k** Relative gene expression of *Sox9 and Comp* in MME$^{-/+}$ FAP derived chondrocytes. **l** Quantification of adipocytes 14 days after transplantation of mT$^+$MME$^{-/+}$ FAPs into glycerol-injected tibialis anterior muscle at the mid belly of the muscle. Related to Fig. 4m. The expression of all genes in **g-k** was normalized to their expression in MME$^-$ FAPs derived white adipocytes (dotted line). Each dot represents a single mouse.Bar graphs represent the mean in **c-f** and represent the mean ± SEM in **g-l**. Student's t-test (two tailed, paired, *p<0.05, **p<0.01, ns>0.05) was used in **c-f**, **i**, and **l**. Two-way ANOVA with Šidáks multiple comparison test (ns>0.05) was used in **g**, **h**, **j**, and **k**.

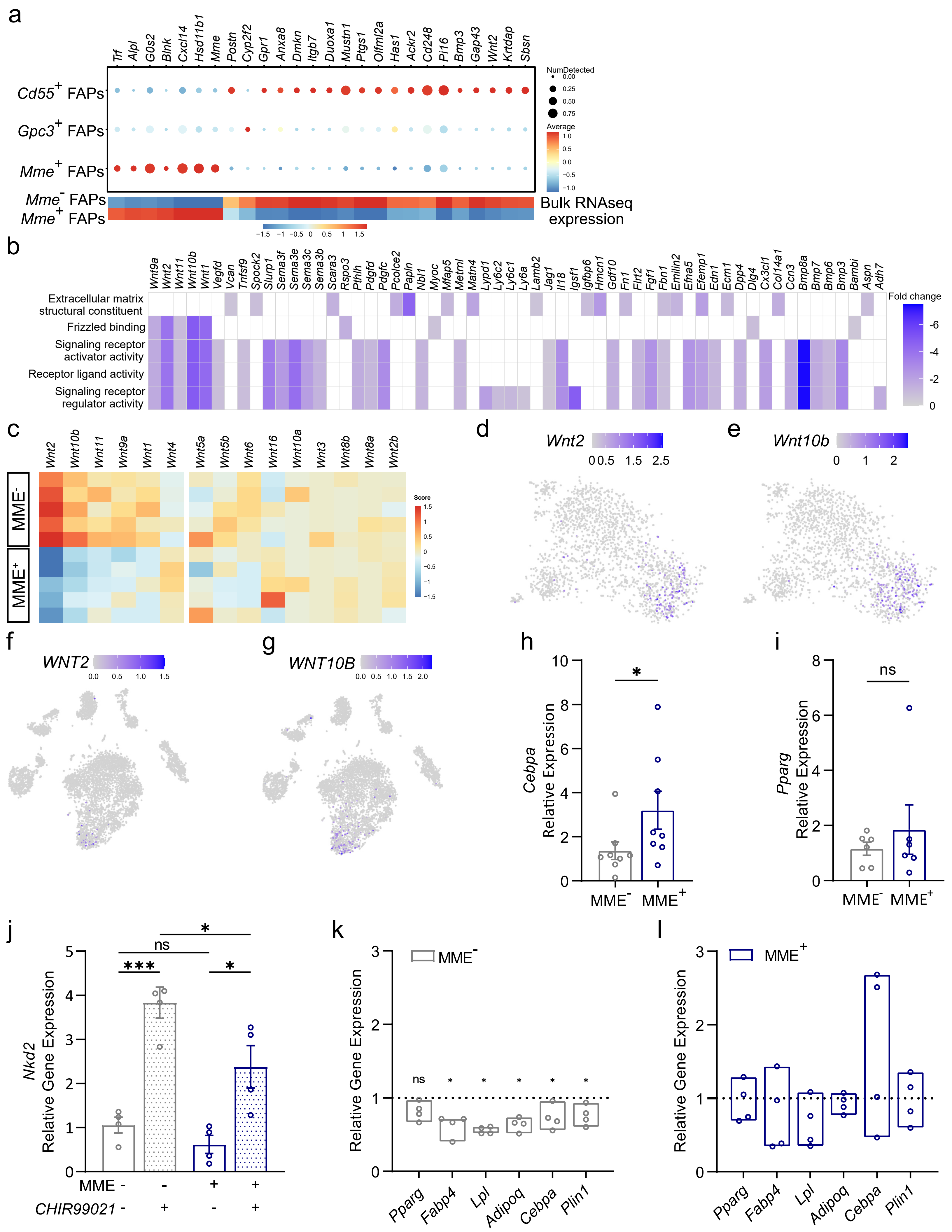

a
Trf Alpl G0s2 Blnk Cxcl14 Hsd11b1 Mme Postn Cyp2f2 Gpr1 Anxa8 Dmkn Itgb7 Duoxa1 Mustn1 Ptgs1 Olfml2a Has1 Ackr2 Cd248 Pi16 Bmp3 Gap43 Wnt2 Krtdap Sbsn
Cd55+ FAPs
Gpc3+ FAPs
Mme+ FAPs
NumDetected
0.00
0.25
0.50
0.75
Average
1.0
0.5
0.0
−0.5
−1.0
Mme- FAPs
Mme+ FAPs
Bulk RNAseq expression
−1.5 −1 −0.5 0 0.5 1 1.5
b
Wnt9a Wnt2 Wnt11 Wnt10b Wnt1 Vegfd Vcan Tnfsf9 Spock2 Slurp1 Sema3f Sema3e Sema3c Sema3b Scara3 Rspo3 Pthlh Pdgfd Pdgfc Pcolce2 Papln Nbl1 Myoc Mfap5 Metrnl Matn4 Lypd1 Ly6c2 Ly6c1 Ly6a Lamb2 Jag1 Il18 Igsf1 Igfbp6 Hmcn1 Gdf10 Fn1 Flrt2 Fgf1 Fbn1 Emilin2 Efna5 Efemp1 Edn1 Ecm1 Dpp4 Dlg4 Cx3cl1 Col14a1 Ccn3 Bmp8a Bmp7 Bmp6 Bmp3 Bambi Aspn Adh7
Extracellular matrix structural constituent
Frizzled binding
Signaling receptor activator activity
Receptor ligand activity
Signaling receptor regulator activity
Fold change
-6
-4
-2
0
c
Wnt2 Wnt10b Wnt11 Wnt9a Wnt1 Wnt4 Wnt5a Wnt5b Wnt6 Wnt16 Wnt10a Wnt3 Wnt8b Wnt8a Wnt2b
MME-
MME+
Score
1.5
1
0.5
0
−0.5
−1
−1.5
d
Wnt2
0 0.5 1.5 2.5
e
Wnt10b
0 1 2
f
WNT2
0.0 0.5 1.0 1.5
g
WNT10B
0.0 0.5 1.0 1.5 2.0
h
Cebpa Relative Expression
10
8
6
4
2
0
*
MME- MME+
i
Pparg Relative Expression
8
6
4
2
0
ns
MME- MME+
j
Nkd2 Relative Gene Expression
5
4
3
2
1
0
***
ns
*
*
MME - - + +
CHIR99021 - + - +
k
MME-
Relative Gene Expression
3
2
1
0
ns * * * * *
Pparg Fabp4 Lpl Adipoq Cebpa Plin1
l
MME+
Relative Gene Expression
3
2
1
0
Pparg Fabp4 Lpl Adipoq Cebpa Plin1

Supplementary Fig 6: MME^{+} FAPs are characterized by reduced WNT signaling and refractory to WNT-mediated inhibition of adipogenesis. Related to Fig. 5.

a Dot plot from mouse scRNA-seq of high variable genes identified in the mouse bulk RNA-seq (see Fig. 5b). Each row represents a FAP subpopulation and each column a gene. Color and size of the dots indicate the level of expression and the percentage of cells that express the gene respectively. The simplified heatmap below displays Z-scores averaged per cell type from the bulk RNA-seq data set (see Fig. 5b). **b** Heatmap showing the top 5 significantly downregulated *molecular function* processes (adjusted p-value < 0.05)in a Gene Ontology (GO) enrichment analysis in *MME*$^{+}$ FAPs from the mouse bulk RNA-seq. Color- coded by the log2-FoldChange respective to *MME*$^{-}$ FAPs. Each row represents a *molecular function* process and each column a gene which is significantly regulated within our dataset that belongs to that *molecular function* process (log2-FoldChange > 0.5 and FDR < 0.05). Blank tiles indicate that a gene does not belong to that *molecular function* process. **c** Heatmap with Z scores showing WNT ligands in MME$^{-/+}$ FAPs. Genes displayed left of the line break are differentially expressed (log2- FoldChange > 0.5 and FDR < 0.05). **d** TSNE plot of *Wnt2* color-coded by logcounts values in mouse FAPs. **e** TSNE plot of *Wnt10b* color-coded by logcounts values in mouse FAPs. **f** TSNE plot of *WNT2* color-coded by logcounts values in FAPs in human muscle. **g** TSNE plot of *WNT10B* color-coded by logcounts values in FAPs in human muscle. **h-i** Relative expression of *Cebpa* (**h**) and *Pparg* (**i**) in freshly sorted MME$^{-/+}$ FAPs. **j** Relative gene expression of *Nkd2* in MME^{-} FAP derived and MME^{+} FAP derived adipocytes after differentiation in low insulin medium with (CHIR99021) or without (Vehicle, DMSO) GSK-3 inhibition. **k** Gene expression of *Pparg, Fabp4, Lpl, Adipoq, Cebpa,* and *Plin1* in MME^{-} FAP derived adipocytes after differentiation in low insulin medium with (CHIR99021) GSK-3 inhibitionas compared to their expression in MME^{-} FAP-derived adipocytes without (Vehicle, DMSO) GSK-3 inhibition (dotted line). **l** Gene expression of *Pparg, Fabp4, Lpl, Adipoq, Cebpa,* and *Plin1* in MME^{+} FAP derived adipocytes after differentiation in low insulin medium with (CHIR99021) GSK-3 inhibitionas compared to their expression in MME^{+} FAP-derived adipocytes without (Vehicle, DMSO) GSK-3 inhibition (dotted line). Each dot represents a single mouse. Bar graphs represent the mean ± SEM. Student's t-test (two tailed, paired, ns>0.05, *p<0.05) was used in **h** and **i**.

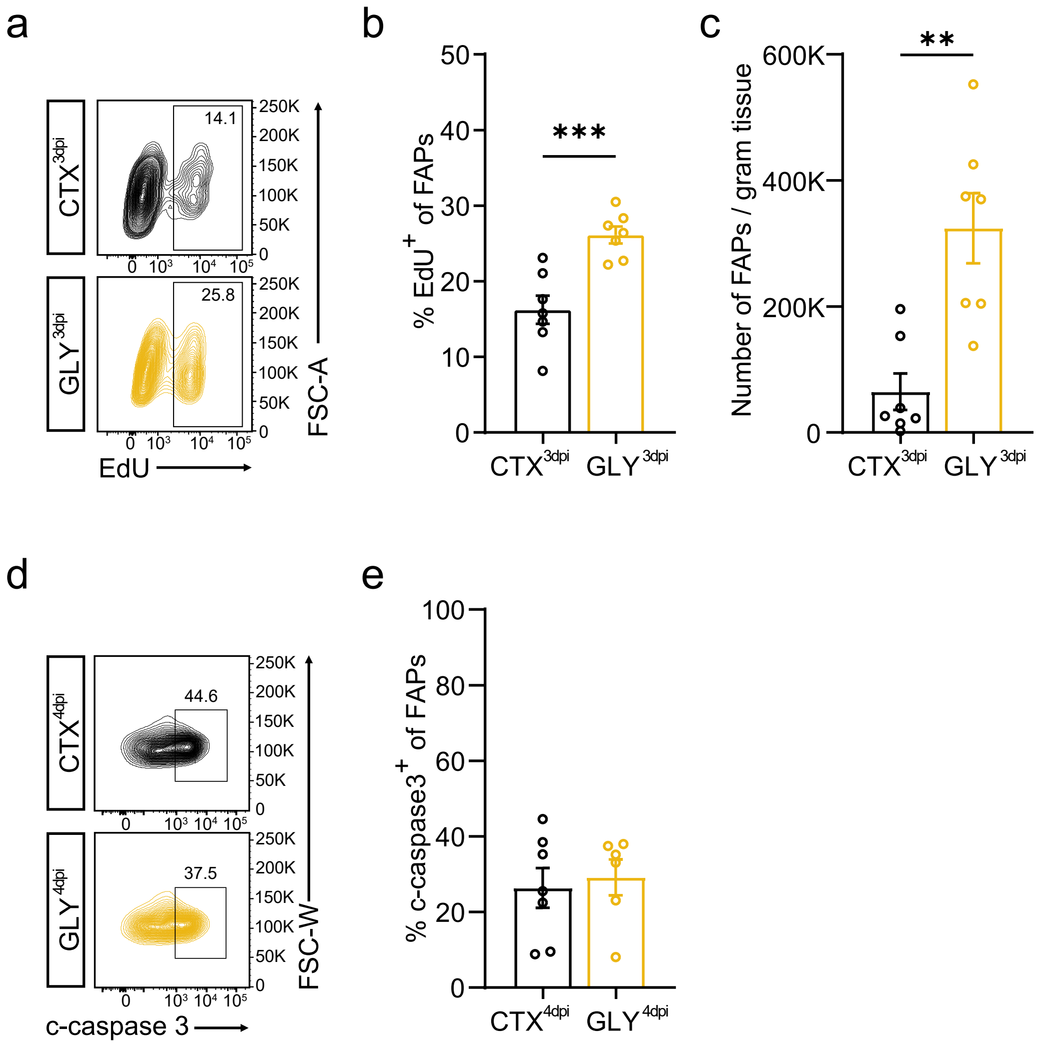
a
CTX3dpi
GLY3dpi
14.1
25.8
EdU
FSC-A
b
% EdU+ of FAPs
***
CTX3dpi
GLY3dpi
c
Number of FAPs / gram tissue
**
CTX3dpi
GLY3dpi
d
CTX4dpi
GLY4dpi
44.6
37.5
c-caspase 3
FSC-W
e
% c-caspase3+ of FAPs
CTX4dpi
GLY4dpi

Supplementary Fig. 7: MME$^+$ FAPS are more prone to apoptosis after injury and deplete upon glycerol injury. Related to Fig. 6.

a-b Representative flow cytometric analysis (**a**) and quantification (**b**) of EdU$^+$ FAPs as a percentage of all FAPs at 3 dpi in cardiotoxin (CTX) injected or glycerol (GLY) injected muscle. **c** Quantification of the number of FAPs in CTX and GLY injected muscle at 3 dpi. **d-e** Representative flow cytometric analysis (**d**) and quantification (**e**) of c-caspase3$^+$ FAPs as a percentage of eGFP$^+$ FAPs at 4 dpi in CTX injected and GLY injected muscle. Each dot represents a single mouse. Bar graphs represent the mean ± SEM. Student's t-test (two tailed, unpaired, $^*p < 0.05$, $^{**}p < 0.01$, $^{***}p < 0.001$) was used in **b,c,** and **e.**

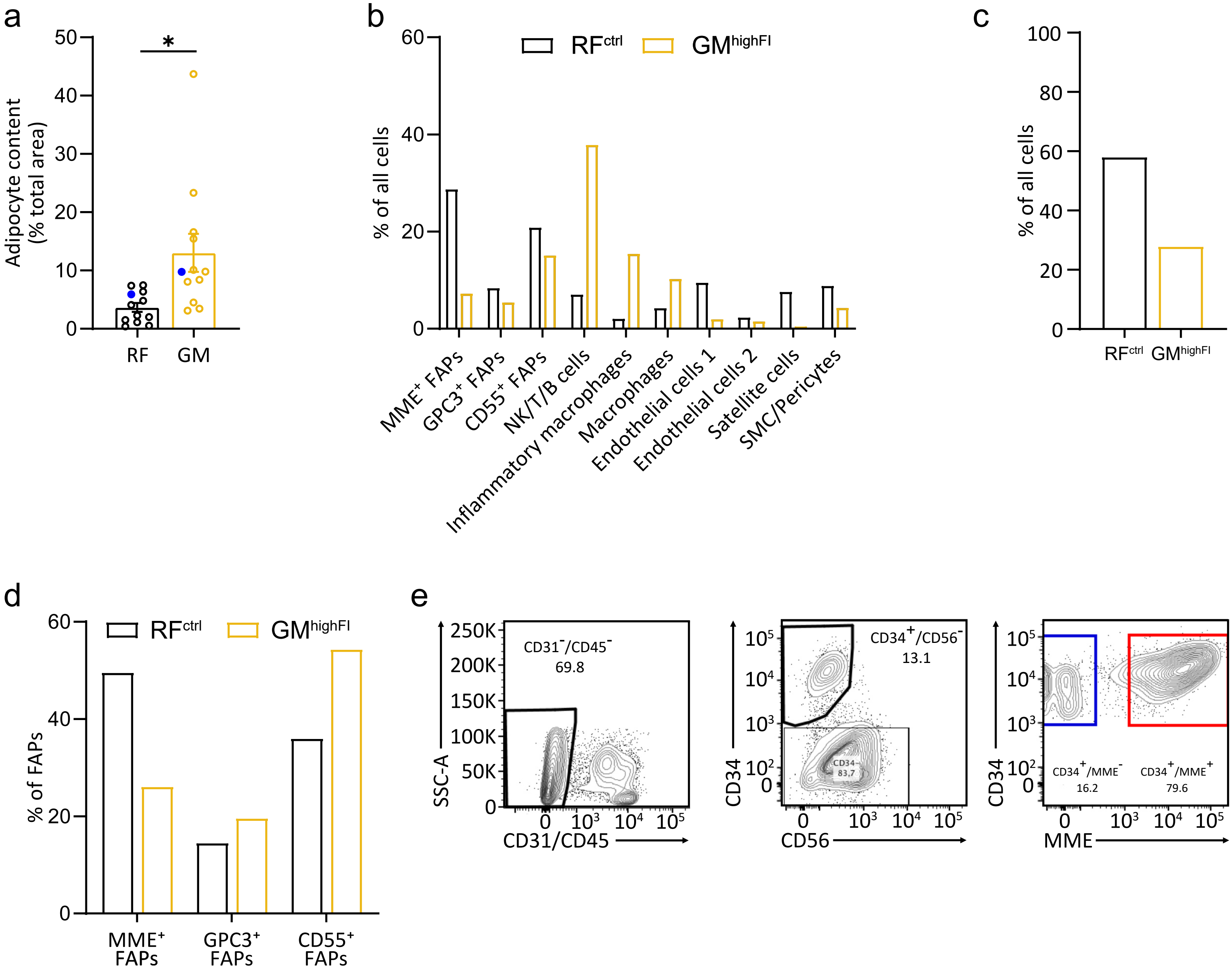

a
Adipocyte content (% total area)
*
RF
GM
b
RFctrl
GMhighFI
% of all cells
MME+ FAPs
GPC3+ FAPs
CD55+ FAPs
NK/T/B cells
Inflammatory macrophages
Macrophages
Endothelial cells 1
Endothelial cells 2
Satellite cells
SMC/Pericytes
c
% of all cells
RFctrl
GMhighFI
d
RFctrl
GMhighFI
% of FAPs
MME+ FAPs
GPC3+ FAPs
CD55+ FAPs
e
CD31-/CD45-
69.8
SSC-A
CD31/CD45
CD34+/CD56-
13.1
CD34-
83,7
CD34
CD56
CD34+/MME-
16.2
CD34+/MME+
79.6
CD34
MME

Supplementary Fig. 8: Human MME^{+} FAPs are highly adipogenic and are exhausted in fatty infiltrated human muscle. Related to Fig. 7.

a Relative adipocyte area as quantified by adipocyte area relative to total biopsy area on H&E-stained sections. Biopsies from the patient used for scRNA-seq are marked with blue dots. **b** Bar plots showing the percentage of the main cell types within each dataset from either control muscle (RF^{ctrl}) or highly fatty infiltrated (GM^{highFI}) human muscle. **c** Bar plots showing the percentage of the combined FAP populations within each dataset from either control muscle (RF^{ctrl}) or highly fatty infiltrated (GM^{highFI}) human muscle. **d** Bar plots showing the percentage of FAPs relative to the total FAP fraction within each dataset from either control muscle (RF^{ctrl}) or highlyfatty infiltrated (GM^{highFI}) human muscle. **e** Representative FACs plots outlining the sorting strategy to isolate MME$^{-/+}$ human FAPs. Each dot represents an individual patient. Bar graphs represent mean ± SEM in **a**. Student's t test (two tailed, paired, *$p<0.05$) was used in **a**. Bar plots in **b, c,** and **d** represent the percentage of all cells or the percentage of FAPs in the RF^{ctrl} and GM^{highFI} single celldatasets.

Supplemental table

Supplementary Table 1. Primers used for RT-PCR. Related to Fig. 5 and Supplementary Fig. 5,6.

Gene Name	Forward Primer	Reverse Primer
Rna18s5	AGTCCCTGCCCTTTGTACACA	CGATCCGAGGGCCTCACTA
Wnt2	ACAACAGAGCTGGAAGGAAGGCTGT	AGTGAAGCCAGTGCCATCCTGG
Wnt10b	GCTGACTGACTCGCCCACCG	AAGCACACGGTGTTGGCCGT
Mme	GGGAGGCTTTATGTGGAAGC	CCGGATTTGTGCAATCAAGT
Lum	CCCACCCTGACAGAGTTCAC	ATTGGCCACTGACACTACCG
Fbn1	TGAGAGTCCGAGCCGCTAGT	ACAGCTTTCTTCTCCAGGGAC
Cd55	GGAGAGCCTAACACAGGTGG	TCTTCGTAACTCTTCGTTGGCT
Lep	TCAAGACCATTGTCACCAGG	TGAAGCCCAGGAATGAAGTC
Adipoq	TGTTCCTCTTAATCCTGCCCA	CCAACCTGCACAAGTTCCCTT
Ucp1	GGACGACCCCTAATCTAATGAG	GCAAAACCCGGCAACAAG
Plin5	CCATCTCGCCTATGAACACTCTT	CAGCTGGGCCAGCATCTC
Plin1	CTGTGTGCAATGCCTATGAGA	CTGGAGGGTATTGAAGAGCCG
Pparg	TCGCTGATGCACTGCCTATG	GAGAGGTCCACAGAGCTGATT
Acta2	GTCCCAGACATCAGGGAGTAA	TCGGATACTTCAGCGTCAGGA
Bglap2	CTGACCTCACAGATCCCAAGC	TGGTCTGATAGCTCGTCACAAG
Runx2	CCTGAACTCTGCACCAAGTCCT	TCATCTGGCTCAGATAGGAGGG
Sox9	AGTACCCGCATCTGCACAAC	ACGAAGGGTCTCTTCTCGCT
Comp	ACTGCCTGCGTTCTAGTGC	CGCCGCATTAGTCTCCTGAA
Cebpa	GCCGAGATAAAGCCAAACAAC	GACCCGAAACCATCCTCTG
Nkd2	GCTCTCAGCTCGGGATTCAC	CCCCTTCGGGACTCTCTCTC
Axin2	GGCTGCGCTTTGATAAGGTC	GCTCATGTGAGCCTCCTCTC
