# Supplementary material for: MME+ fibro-adipogenic progenitors are the dominant adipogenic population during fatty infiltration in human skeletal muscle
Source: Commun Biol. 2023 Jan 27;6:111. doi: 10.1038/s42003-023-04504-y (PMC9883500; doi:10.1038/s42003-023-04504-y)
Supplement: Supplementary file 5 — Reporting summary [file 42003_2023_4504_MOESM5_ESM.pdf]

nature portfolio

Corresponding author(s): COMMSBIO-22-3502B

Last updated by author(s): 20/12/2022

Reporting Summary

Nature Portfolio wishes to improve the reproducibility of the work that we publish. This form provides structure for consistency and transparency in reporting. For further information on Nature Portfolio policies, see our Editorial Policies and the Editorial Policy Checklist.

Statistics

For all statistical analyses, confirm that the following items are present in the figure legend, table legend, main text, or Methods section.

n/a	Confirmed	
☐	☒	The exact sample size (n) for each experimental group/condition, given as a discrete number and unit of measurement
☐	☒	A statement on whether measurements were taken from distinct samples or whether the same sample was measured repeatedly
☐	☒	The statistical test(s) used AND whether they are one- or two-sided<br>*Only common tests should be described solely by name; describe more complex techniques in the Methods section.*
☒	☐	A description of all covariates tested
☐	☒	A description of any assumptions or corrections, such as tests of normality and adjustment for multiple comparisons
☐	☒	A full description of the statistical parameters including central tendency (e.g. means) or other basic estimates (e.g. regression coefficient) AND variation (e.g. standard deviation) or associated estimates of uncertainty (e.g. confidence intervals)
☐	☒	For null hypothesis testing, the test statistic (e.g. F, t, r) with confidence intervals, effect sizes, degrees of freedom and P value noted<br>*Give P values as exact values whenever suitable.*
☒	☐	For Bayesian analysis, information on the choice of priors and Markov chain Monte Carlo settings
☒	☐	For hierarchical and complex designs, identification of the appropriate level for tests and full reporting of outcomes
☒	☐	Estimates of effect sizes (e.g. Cohen's d, Pearson's r), indicating how they were calculated

Our web collection on statistics for biologists contains articles on many of the points above.

Software and code

Policy information about availability of computer code

Data collection	Fluorescence imaging: ZEN software (Zeiss). Confocal imaging: FV31S-SW. Flow cytometry: FACS Diva
Data analysis	Bioinformatic analysis: CellRanger 6.0.2, Kallisto, R 4.1.2, R packages: scater 1.22.0, scran 1.22.0, harmony 0.1.0, Seurat 4.0.5, SingleR 1.8.0, DESeq2 1.34.0, fgsea 1.20.0, clusterProfiler 4.2.0. Image analysis: ImageJ 1.53. Flow cytometry analysis: FlowJo 10.4.2. Statistical analysis: GraphPad Prism 9

For manuscripts utilizing custom algorithms or software that are central to the research but not yet described in published literature, software must be made available to editors and reviewers. We strongly encourage code deposition in a community repository (e.g. GitHub). See the Nature Portfolio guidelines for submitting code & software for further information.

Data

Policy information about availability of data

All manuscripts must include a data availability statement. This statement should provide the following information, where applicable:

- Accession codes, unique identifiers, or web links for publicly available datasets
- A description of any restrictions on data availability
- For clinical datasets or third party data, please ensure that the statement adheres to our policy

All sequencing data (single-nuclei, single-cell, and bulk RNA-seq) reported in this study are available at the Gene Expression Omnibus (GEO) repository under the accession number GSE200487. Please check https://shiny.debocklab.hest.ethz.ch/Fitzgerald-et-al/ for data visualization. All other data are available from the corresponding author on request.

Field-specific reporting

Please select the one below that is the best fit for your research. If you are not sure, read the appropriate sections before making your selection.

☒ Life sciences ☐ Behavioural & social sciences ☐ Ecological, evolutionary & environmental sciences

For a reference copy of the document with all sections, see nature.com/documents/nr-reporting-summary-flat.pdf

Life sciences study design

All studies must disclose on these points even when the disclosure is negative.

Sample size	No sample-size calculations were performed. Sample size was determined to be adequate based on the magnitude and consistency of measurable differences between groups.
Data exclusions	Grubbs test was used to determine significant outliers (Alpha = 0.05).
Replication	Number of replicates are explained in the figure legends.
Randomization	Mouse experiments: for mouse experiments with different treatments (CTX or glycerol) mice were randomly assigned to treatment groups.
Blinding	Flow cytometry analysis: investigators were blinded during the experiment. Cell experiments: investigators were not blinded to cell type during preparatory cell culture and cell seeding. Transplantation experiments: investigators were not blinded during injections but were blinded during image analysis. Bioinformatic analysis: investigators were not blinded during bioinformatic analyses.

Reporting for specific materials, systems and methods

We require information from authors about some types of materials, experimental systems and methods used in many studies. Here, indicate whether each material, system or method listed is relevant to your study. If you are not sure if a list item applies to your research, read the appropriate section before selecting a response.

Materials & experimental systems

n/a	Involved in the study	
☐	☒	Antibodies
☒	☐	Eukaryotic cell lines
☒	☐	Palaeontology and archaeology
☐	☒	Animals and other organisms
☐	☒	Human research participants
☒	☐	Clinical data
☒	☐	Dual use research of concern

Methods

n/a	Involved in the study	
☒	☐	ChIP-seq
☐	☒	Flow cytometry
☒	☐	MRI-based neuroimaging

Antibodies

Antibodies used	Human CD45-FITC (12µl/sample, 130-114-567, Clone 5B1, Miltenyi Biotec), Human CD31-FITC (4µl/sample, 130-110-668, Clone REA730, Miltenyi Biotec), Human MME-PE (4 µl/sample, Clone REA877, 130-114-693, Miltenyi Biotec), Human CD56-BV421 (1:100, 562752, BD Bioscience), Human CD82-PE-vio770 (130-101-302 10µl/sample, Miltenyi Biotec), Human CD34-APC (20µl/sample, clone 581, BD Bioscience), Human Perilipin-1 (rabbit, 1:200, Cell Signaling Technologies), Human alpha-smooth muscle actin (1:150, 593 A5228, Sigma), Alexa-fluor 647 goat-anti-mouse, Alexa-fluor 568 goat-anti-rabbit (1:500; A-21235 and A-11011, Invitrogen, Thermo Fisher Scientific), anti-mouse MME antibody (Mouse Neprilysin/CD10 Antibody, R&D Systems AF1126; 1:200), Non phospho (Active) β Catenin (Ser33/37/Thr41) Antibody (#4270, Cell Signaling), anti-Perilipin A primary antibody (P1998, Sigma-Aldrich), Anti-human MME antibody (AF1182, R&D Systems), biotinylated anti-cleaved caspase 3 (9654S, Cell Signaling Technology)
Validation	Single color samples and fluorescence minus one (FMO) controls were used for all antibodies.

Animals and other organisms

Policy information about studies involving animals; ARRIVE guidelines recommended for reporting animal research

Laboratory animals	B6.129S4-Pdgfratm11(EGFP)Sor/J<br>B6.129S4-Pdgfratm11(EGFP)Sor/J x Gt(ROSA)26Sortm4(ACTB-tdTomato,-EGFP)Luo/J
Wild animals	*Provide details on animals observed in or captured in the field; report species, sex and age where possible. Describe how animals were caught and transported and what happened to captive animals after the study (if killed, explain why and describe method; if released, say where and when) OR state that the study did not involve wild animals.*

Field-collected samples	*For laboratory work with field-collected samples, describe all relevant parameters such as housing, maintenance, temperature, photoperiod and end-of-experiment protocol OR state that the study did not involve samples collected from the field.*
Ethics oversight	All animal procedures were approved by the Veterinary office of the Canton of Zurich, Switzerland (license nr, ZH-180/18, ZH094-17).

Note that full information on the approval of the study protocol must also be provided in the manuscript.

Human research participants

Policy information about studies involving human research participants

Population characteristics	The inclusion criteria were end-grade HOA based on clinical and radiological examinations, scheduled for THA, men, and age between 55 and 75 years. Exclusion criteria were pain on the contralateral hip, lower limb surgeries in the previous 10 years, inability to walk without aids, BMI > 35 kg*m-2, and cardiorespiratory diseases. 12 men were recruited for this study (67 ± 6yrs, 84 ± 10kg, 179 ± 4cm).
Recruitment	Patients were recruited from the Department of Hip Orthopaedic Surgery, Schulthess Clinic (Zurich, Switzerland).
Ethics oversight	All patients signed an informed consent before participating in the study. The study was conducted according to the Declaration of Helsinki and the protocol was approved by the Ethics Committee of the Canton of Zurich (KEK number: 2016-01852).

Note that full information on the approval of the study protocol must also be provided in the manuscript.

Flow Cytometry

Plots

Confirm that:

- [x] The axis labels state the marker and fluorochrome used (e.g. CD4-FITC).
- [x] The axis scales are clearly visible. Include numbers along axes only for bottom left plot of group (a 'group' is an analysis of identical markers).
- [x] All plots are contour plots with outliers or pseudocolor plots.
- [x] A numerical value for number of cells or percentage (with statistics) is provided.

Methodology

Sample preparation	Cells were isolated via tissue enzymatic digestion and incubated with antibodies on ice prior to analysis. Nuclei were isolated via Dounce homogenization and detergent application.
Instrument	Flow cytometry was performed on a BD LSRFortessa instrument. FACS sorting was performed on a BD FACSAria Fusion, FACS-AriaIII, or Sony SH800S sorter.
Software	Collection: Data was collected using FACS Diva software for BD instruments and SH800S software was used for the Sony instrument
Cell population abundance	For human cell sorting experiments the purity of the populations was checked following the sort by rerunning the samples. For mouse sample purity, cell populations were checked by qPCR where the MME+ population showed 30X higher expression of MME than the MME- population. Furthermore, MME was one of the top 10 high variable genes in the MME+ population in the bulk sequencing of sorted MME+/- FAPs.
Gating strategy	For mouse flow cytometry analysis, debris was initially eliminated using FSC/SSC and singlets identified using FSC-A vs FSC-H strategy. Cells were further delineated from debris using hoechst staining. All further gates were determined using FMO controls. For human and mouse cell sorting, example gating strategy is provided.

- [ ] Tick this box to confirm that a figure exemplifying the gating strategy is provided in the Supplementary Information.
